# Fructose metabolism in humans – what isotopic tracer studies tell us

**DOI:** 10.1186/1743-7075-9-89

**Published:** 2012-10-02

**Authors:** Sam Z Sun, Mark W Empie

**Affiliations:** 1 Compliance, Archer Daniels Midland Company, 1001 North Brush College Road, Decatur, IL, 62521, USA

**Keywords:** Fructose, Glucose, Isotope tracer, Metabolism

## Abstract

Fructose consumption and its implications on public health are currently under study. This work reviewed the metabolic fate of dietary fructose based on isotope tracer studies in humans. The mean oxidation rate of dietary fructose was 45.0% ± 10.7 (mean ± SD) in non-exercising subjects within 3–6 hours and 45.8% ± 7.3 in exercising subjects within 2–3 hours. When fructose was ingested together with glucose, the mean oxidation rate of the mixed sugars increased to 66.0% ± 8.2 in exercising subjects. The mean conversion rate from fructose to glucose was 41% ± 10.5 (mean ± SD) in 3–6 hours after ingestion. The conversion amount from fructose to glycogen remains to be further clarified. A small percentage of ingested fructose (<1%) appears to be directly converted to plasma TG. However, hyperlipidemic effects of larger amounts of fructose consumption are observed in studies using infused labeled acetate to quantify longer term de novo lipogenesis. While the mechanisms for the hyperlipidemic effect remain controversial, energy source shifting and lipid sparing may play a role in the effect, in addition to de novo lipogenesis. Finally, approximately a quarter of ingested fructose can be converted into lactate within a few of hours. The reviewed data provides a profile of how dietary fructose is utilized in humans.

## Introduction

Fructose has been a part of the human diet for many thousands of years, and it is found in highest concentrations in fruits and to a lesser degree in vegetables. Cane, beet, and corn sugars are produced industrially, and their use results in significant quantities of added sugars entering the diet, about half of which is fructose [1]. Cane and beet sugars are comprised of the disaccharide sucrose (glucose bonded to fructose) and are commonly called table sugar or simple sugar. Corn sugars come from corn starch, and mainly consist of high fructose corn syrup 55 (HFCS 55; 55% fructose-41% glucose), HFCS 42 (42% fructose-52% glucose), and corn syrup (glucose and oligoglucose with trace amounts of fructose). During the last several decades, the prevalence of obesity and metabolic syndrome has risen dramatically on a global basis, but more so in the U.S. population. Because the prevalence is chronologically and statistically correlated with the increase of added sugar intakes, particularly HFCS in the U.S. (HFCS is not consumed significantly outside the U.S.), some have proposed the intake of HFCS or fructose as a free monosaccharide may be a cause of various adverse health consequences [2]. Conventional clinical trials and ecological studies have been conducted to assess the hypotheses, but not all results are found to be supportive. Conventional studies often cannot reveal details of interconnecting metabolic pathways when testing fructose or fructose-containing sugars, but they also cannot clearly distinguish a mechanistic cause associated with an observed physiological consequence linked to the sugar consumed. This is because the ordinary diet contains multiple forms of saccharides which are inter-convertible in the body and share many steps of the carbohydrate metabolism pathways.

Over the last decade, a series of controversies have arisen regarding fructose consumption. In 2004, a commentary was written hypothesizing that the “high” fructose content in HFCS was the cause of the obesity rise in America [3]. This was based on the association of the obesity prevalence rise with the replacement of cane and beet sugar by HFCS, even though the fructose content of these two sweetener sources is essentially the same. Later, several dietary studies using calorically high doses of fructose were published to investigate fructose modulation of leptin hormone status, with a suggestion that chronic changes in this hormone level could lead to weight gain [4,5]. However, other studies and evidence based reviews do not always support these findings [6-13]. Recently, Welsh et al. [14] reported that the intake of added sugar has significantly decreased between 1999 and 2008 while the obesity prevalence has continued to rise. The current view is that obesity is a matter of energy balance [15,16]. Next, the fructose moiety in sugars was hypothesized to cause high serum uric acid which could lead to the development of Type-2 diabetes [17]. There is currently no direct proof for a cause and effect relation of urate with diabetes, and NHANES data suggests no relation of serum urate with fructose intake at ordinary dietary consumption levels [18]. Then, another hypothesis has been raised that dietary fructose may potentially lead to Non Alcoholic Fatty Liver Disease (NAFLD) and augmented de-novo triglyceride synthesis, based on an analysis of hormone regulated lipid pathways in the liver [19,20]. It is known that high dietary levels of fructose can increase serum triglycerides. However, all the factors linked to the development of fatty liver disease are not well understood and can include, insulin resistance, inflammation, fat re-deposition, abnormalities in control of reactive oxygen species [21] and uncoupling proteins in mitochondria [22]. NAFLD is currently an important and actively researched field relative to dietary sugar intakes.

Additionally, it is important to understand the practical significance of testing an effect from a single sugar using an unrepresentative dose compared to the true population sugar intake, a question which is currently under debate [23-26]. In many of the intervention studies involved with studying the various hypotheses mentioned above, very high doses of sugars over short term were often applied, the study designs were more similar to toxicological studies, and the studies were only able to draw associative conclusions between applied dose and observed health-related outcomes in the subjects studied. The observed biological changes, although statistically significant by a P-value ruling, were often only fluctuations within normal ranges. These studies rarely measured actual development of disease or the intermediate metabolites characterizing mechanism-based reactions. To begin to prove true effect of a diet component, it is useful to study the component disposal through the common central pathways at the molecular level. These studies are facilitated and detailed by the use of isotope tracer labeled precursors, and this concept is the stimulus for this review.

**Figure 1:**
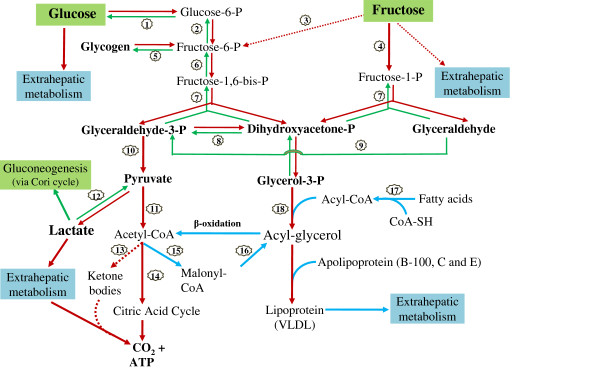
**Major metabolic pathways and flux of dietary glucose and fructose.** P = phosphate. For enzymes numbered in circles: 1 = hexokinase/glucokinase or Glucose-6-phosphatase, 2 = phosphoglucose isomerase, 3 = hexokinase, 4 = fructokinase, 5 = glycogen synthase or phosphorylase, 6 = phosphofructokinase, 7 = aldolase, 8 = triose phosphate isomerase, 9 = triose kinase, 10 = several enzymes including pyruvate kinase, 11 = pyruvate dehydrogenase complex,12 = lactate dehydrogenase,13 = ketothiolase and other 3 enzymes,14 = enzyme group related to citric acid cycle, 15 = acetyl CoA carboxylase,16 = multienzyme complexes, 17 = acyl CoA synthase, 18 = glycerol-phosphate acyl transferase and triacylglycerol synthase complex. The dashed-line and arrow represents minor pathways or will not occur under a healthy condition or ordinary sugar consumption. The compound names in **bold** would be major metabolic intermediates or end products of glucose or fructose metabolism.

The questions raised by the above hypotheses reach into the broader metabolome and fluxome. Our understanding of the metabolism of glucose and fructose as separate sugars is founded upon many years of study, and detailed anabolic and catabolic pathways are known [27]. Recently, the extended metabolism of glucose and fructose has been reviewed by Tappy and Le [28]. Glucose and fructose carbons are utilized through the glycolysis, gluconeogenesis, glycogenolysis, tricarboxylic acid (TCA) cycle, lactate production (Cori cycle), pentose phosphate shunt, and lipid synthesis pathways in various physiological compartments to provide substrates for glycogen homeostasis, amino acids, other sugars, fats and energy (e.g. ATP). Glucose and fructose enter the metabolic pathways differently (Figure 1), with glucose being converted to 1,6-diphosphorylated fructose before being cleaved into the three carbon metabolic intermediates, dihydroxy acetone phosphate and glyceraldehyde 3-phosphate. Absorbed fructose is only mono-phosphorylated before being cleaved into glyceraldehyde and dihydroxy acetone phosphate, which is the common intermediate with the glucose pathway. Glucose utilization can be regulated before cleavage, whereas fructose is less regulated. This initial difference has prompted some to hypothesize that, because fructose cleavage by-passes key feedback regulatory steps in the glucose metabolic pathway, this bypass may lead to increases of fatty acid synthesis, which may contribute to causes of obesity [4]. This hypothesis relies on a simplified metabolic pathway analysis and on studies using pure fructose in comparison to pure glucose, a situation which rarely occurs in the American diet [29,30].

In nature, fructose commonly occurs together with glucose, and composition values for some foods have been tabulated by the USDA on its website: http://www.nal.usda.gov/fnic/foodcomp/search/. The metabolism of food derived sucrose, fruit sugars, honey, and high fructose corn syrup, major sources of fructose and glucose in the diet, are currently under study, and the biological effects resulting from the use of experimentally formulated mixtures of glucose and fructose are relevant to our understanding. The use of mixed sugars are more metabolically predictive of dietary consequences than that from single monosaccharides studied individually, as metabolism of each type of sugar is not independent from the other (discussed below). Metabolic interactions between glucose and fructose significantly impact general sugar metabolism.

Owing to the complexity of fructose and glucose metabolism, conventional feeding study approaches are usually less informative than isotope tracer studies for obtaining a clear picture of mechanisms for utilization of dietary fructose or glucose. It is known that carbon moieties in fructose and glucose can be inter-converted in the liver [31], and thus studying the disposal and metabolic effects of these dietary sugars with respect to one another is most definitively conducted using isotope-labeled sugars as tracers. A number of these isotopic tracer studies exist, and many are found in the literature dated before the year 2000. Although all pathways have not been completely studied for fructose disposal and metabolism under different physiological conditions, a significant number of reports on fructose isotope tracer studies are published. In this work, we have reviewed fructose disposal and metabolism in humans based on isotope tracer studies to better understand from a molecular stand point fructose oxidation, fructose conversion into glucose, fructose conversion into lipids, and fructose conversion into lactate.

## Method

Pubmed and Scopus websites were searched using 2 or more key word combinations of fructose, glucose, sucrose, tracer, ^13^C, ^14^C, and isotope with limitation of using human studies. When reviewing the metabolic fate of dietary fructose (including oxidation, glucose conversion, glycogen synthesis, lipid conversion and lactate production), the data were obtained from publications that met the following criteria: adult subjects, unbound or bound fructose studied, isotope-tracer used, in English, and with metabolic-related study purposes. In total, 34 papers met the criteria. Other conditions related to study design were not used as exclusion criteria, such as subject fasting status, ways of fructose administration, and sample size. Many of the studies on fructose oxidation were conducted by researchers interested in exercise and athletic performance enhancement. In some studies, fructose ingestions were combined with glucose or with other nutrient intravenous infusion. Dose levels of fructose and administration methods (bolus or several small portions) also varied between studies. As a result, there is significant heterogeneity between studies and the protocol, and quality among the tracer studies cited in this review may not be similar. The expired CO_2_ recovery coefficients, correction factors (k factor) for the collection loss of expired CO_2_, were not identical in the studies investigating fructose oxidation. This may account for some of the observed variations in oxidation rates of ingested fructose [32].

The following sections will review what tracer studies tell us about the disposal and metabolism of dietary fructose as a single sugar or as a mixture with glucose, either bound or free. In this way, the origins and fate of specific carbons in these sugars may be determined in relation to their partition among metabolic pathways and how the presence of one sugar influences the metabolism of the other. In two sections, studies are included which use non-labeled fructose together with labeled pathway compounds to assess the impact of ingested fructose on metabolites or pool intermediates leading to alterations in relevant end points, such as glucose production or de novo lipogenesis. In this way, fructose carbons themselves are not followed, but broader anabolic responses affected by the fructose load can be measured. This isotopic method makes use of Mass Isotopomer Distribution Analysis (MIDA), a technique reviewed by Hellerstein and Neese (1999) [33]. Although these studies investigate a number of other important physiological parameters, we report here only the results which are directly measured by the tracer itself.

### Fructose absorption

To place the metabolism of labeled sugars in context, it is helpful to briefly discuss what is known about the uptake of fructose, glucose and sucrose from the gut, interdependencies, and entry into the circulation. These considerations should be taken into account when designing studies. A number of the studies discussed in this section do not use tracer labeled sugars, but are included to provide a comprehensive description. The absorption rate of fructose alone from the small intestine is slower than that of glucose. This is partly due to the differences in the absorption process between the two monosaccharides. Glucose is absorbed from the intestine into the plasma via more than one active glucose co-transporter protein. SGLT1 transports glucose from the intestinal lumen through the apical membrane into the intestinal epithelial cells. Exit from the epithelial cells through the basolateral membrane to the blood is facilitated by GLUT2. Fructose is absorbed at a slower rate from the lower part of duodenum and jejunum both passively and actively by the brush-border membrane transporter 5 (GLUT-5) and transported into blood also by GLUT2 [34,35]. GLUT transporters are primarily made up of 13 multiple homologous proteins (GLUT 1–12 and 14) and they are located throughout the body often exhibiting tissue specificities [36]. The capacity for fructose absorption in humans is not completely clear, but early studies suggested that fructose absorption is quite efficient, though it is less efficient than that of glucose or sucrose [34]. The slower absorption and prolonged contact time with the luminal intestinal wall would be expected to result in the stimulation of regulatory and satiety signals and release of hormones from enteroendocrine cells [37,38].

When fructose is consumed as the sole carbohydrate source, it can be incompletely absorbed, and as a result, produces a hyperosmolar environment in the intestine. A high concentration of solute within the gut lumen draws fluid into the intestine which can produce feelings of malaise, stomachache or diarrhea [39], and results in decreased food intake. However, when glucose is also present, malabsorption is significantly attenuated [40]. Riby et al. [34] compiled data from five studies comparing glucose, fructose and mixtures of the two for degree of absorption, by measurement of breath hydrogen as an indicator of malabsorption. Pure fructose alone produced dose-dependent evidence of malabsorption starting from12 gram ingestion loads, while glucose and sucrose individually produced no intolerance up to 50 gram ingestion loads. Incremental amounts of added free glucose to a 50 gram fructose load dose-dependently attenuated malabsorption symptoms, and at the equimolar mixture of the two (up to 100 grams total sugars), no malabsorption was observed. Thus, how studies are designed to deliver the various sugars can have an impact on sugar uptake and appearance in the blood.

Sucrose is a valid comparison for glucose-fructose mixtures, as the disaccharide is cleaved by the enzyme sucrase into the mono sugars before being absorbed into the circulation. Comparison of sucrose absorption rates in 32 normal subjects with an equivalent amount of monosaccharide mixture containing glucose and fructose, infused intralumenally to avoid gastric hydrolysis, resulted in the similar absorption rates for each glucose and fructose component of the test [41]. In another study, type-2 diabetic patients were fed sucrose or HFCS with a background diet, resulting in plasma glucose AUC’s not being different between sucrose and the HFCS, nor were mean plasma insulin values [42]. It was also shown that mucosal-to-serosal glucose flux was similar between sucrose and glucose + fructose mixture solution, but rates depended on sucrase and sodium-dependent glucose transport in an in vitro study [43]. Other comparison studies in normal men and women [44] and in diabetics [45,46] produced no differences in intestinal uptake between sucrose and honey (a glucose-fructose mixture). Thus, the body appears to handle oral free glucose-fructose mixtures or HFCS similarly as sucrose and that hydrolysis of sucrose does not appear to be rate limiting for uptake.

Once absorbed, glucose is delivered to the liver then to peripheral organs for utilization, and its entrance into muscle and fat cells is insulin dependent. Fructose is primarily delivered to and metabolized in the liver for energy and for two and three carbon precursor production without dependence on insulin. Bolus or divided doses of 50–150 g fructose produce plasma concentrations of 3–11 mg/dl of this sugar [47-52], while glucose can spike upwards of 150 mg/dl and more. Although little dietary fructose appears in the circulation, it can influence plasma glucose concentrations via sugar inter-conversion. In man, studies indicate fructose to glucose conversion may occur to a highly significant degree (reviewed below) and that this conversion occurs via the 3-carbon intermediate pathways. The extent of inter-conversion may be species dependent.

Key points: 1) Fructose is readily absorbed and its absorption is facilitated by the presence of co-ingested glucose. Sucrose, honey, 50:50 glucose-fructose mixtures and HFCS all appear to be similarly absorbed. 2) Fructose itself is retained by the liver, while glucose is mainly released into the circulation and utilized peripherally. And, 3) Plasma levels of fructose are an order of magnitude (10–50 folds) lower than circulating glucose, and fructose elicits only a modest insulin response. This lower glucose and insulin response by the body to fructose intake has been considered desirable for diabetic diets.

### Fructose and glucose metabolic flux

Fructose and glucose metabolic flux is briefly described in Figure 1. The important point of distinction between glucose and fructose metabolism resides in two areas. Absorbed fructose is extracted by, held, and processed in the liver, with little fructose circulating in the blood stream or delivered to peripheral tissues. Absorbed glucose or that produced in the liver from fructose or other precursors is either metabolized in the liver or exported to the blood stream and further to extrahepatic tissues. Most absorbed fructose is cleaved in the liver into glyceraldehyde and dihydroxy acetone phosphate, and these trioses further go to glycerol phosphate and pyruvate metabolic pathways, respectively. With both fructose and glucose, lactate conversion plays an important role in distributing carbohydrate potential energy between gluconeogenesis and acetyl CoA, with entry into the TCA cycle or use in lipid synthesis (Figure 1) [53,54]. Lactate discharge is also a means for fructose carbons to escape the liver and be transported to peripheral tissues. Fructose cleavage to glyceraldehyde can result in the production of glycerol via reduction. It was observed that blood glycerol concentration increased after fructose ingestion in exercise subjects [55,56]. The noted glycerol increases after fructose ingestion are either greater or similar compared with the values after glucose ingestion, and the produced glycerol can be oxidized for energy. However, the metabolic balance between glycerol produced from fructose and central pathway trioses has not been clearly determined.

Given the complexity and interdependencies of energy metabolism and biochemical synthesis arising from sugars, consideration of the flux of carbons among these pathways is critical to understanding the health consequences of consuming these nutrients. Single sugar distribution and fluxes between pathways are not easily studied without isotopic labels. Classically, a limited number of metabolites are characterized in a study and some disposal points can be missed. More recently, a computational technique is being employed utilizing. Nuclear Magnetic Resonance (NMR) or mass spectral analyses of the ^13^C isotopomer distribution of metabolites, following administration of labeled precursors. These precursors may be uniformly labeled compounds or labeled at specific carbons, depending on the question to be answered. An empirical metabolic flux analysis profile is generated which can be mathematically modeled without being constrained by physical chemistry rigor, as reviewed by Selivanoc and Lee [57,58]. This technique allows one to model metabolite fluxes which may not be well characterized or understood from direct enzymatic or physical chemistry data. A second method is under development to mathematically model general metabolism and interdependencies of pathways using known thermodynamic free energy and kinetic constant parameters for each reaction in the pathway sequences [59], but there is currently insufficient data to apply to fructose metabolism questions.

Each method has advantages and disadvantages, and likely the combination of both is needed for optimal predictive power. In the future, with these tools one should be able to predict outcomes from sugars supply as a function of the organism’s energy (ATP) status, oxidation/reduction potential (NADH/NADPH) and nutrient dependent cofactors. Metabolic differences among compartments and their interactions as a whole should be included. Experimentation should account for metabolome interactions, and study results should be interpreted carefully with respect to the experimental conditions employed.

Key points: 1) Fructose is observed to enter all the pathways of disposal as found for glucose glycolysis and the TCA cycle. 2) Three carbon intermediates provide a means for fructose to be released from the liver and to be utilized peripherally, which suggests that physiological effects observed should be integrated with the co-effect and metabolic fluxes arising from all sugars using these pathways.

### Metabolic fate of dietary fructose

The following reviews the data as presented in the papers. As depicted in Figure 1, the interdependence of metabolic pathways of fructose and glucose can influence the flow of metabolites and their temporal appearance as other compounds. Thus, in discussing the disposal of fructose carbons, e.g. through oxidation, one cannot accurately distinguish if the labeled CO_2_ arose directly from the fructose itself, or from fructose which had undergone conversion to neoformed glucose, neoformed lactate, or other neoformed compounds. Where the conversions occur, these metabolites can be readily transported out of the liver to other tissues, altering the temporal appearance of metabolites. Further complicating the analysis, some studies used fructose labeled with ^13^C at different positions of its carbon backbone, uniformly labeled fructose, or ^13^C naturally enriched fructose. The different labeling may also influence the appearance of the isotope tracer in various metabolites. Using uniformly labeled fructose would limit the potential complications from different labeling positions on isotope tracer appearance in its metabolites. It should also be noted that most of the tracer studies described in the following sections are short-term dietary studies (monitored periods shorter than 8 hours) and may not reflect longer term effects of fructose, such as on de novo lipogenesis, VLDL TG production, or other metabolic specificities.

#### Fructose oxidation

Multiple studies have been conducted to observe how much fructose and other sugars can be oxidized following ingestion. Table 1 summarizes fructose and other sugar oxidation data reported from tracer studies in humans under different experimental designs. In all, 19 relevant studies were found which met the inclusion criteria of this review. The first 4 studies cited in Table 1 used resting subjects with fructose ingestion levels from 0.5-1.0 g/kg body weight (bw). Within the study monitoring periods, the ingested fructose was oxidized from 30.5% to 59%. The study by Chong and colleagues [48] showed fructose was oxidized faster than glucose (30.5% vs 24.5%). This effect may be due to less regulation of phosphorylation for fructose or to a wider tissue distribution of glucose. Oxidation rates increase as the dose increases but would be attenuated by its rate of absorption when intake amounts are large. Delarue et al. [49] indicated when the fructose administration dose increased from 0.5 to 1 g/kg bw, the oxidation amount of fructose correspondingly increased, such that a similar percent of the given fructose dosage was oxidized (56% and 59%, respectively). However, there is a difference in oxidation rates between normal and diabetic subjects, in that normal subjects could more efficiently oxidize fructose than type-2 diabetics (38.5% vs 31.3% of given dosage) [52].

**Table 1:** **Oxidation of Dietary Fructose, Glucose, and Other Sugars in Tracer Studies**^**(1)**^

**Subjects**	**Exercise**	**Hours**	**Sugar dosage (g)**	**Tracer**	**Oxidation**	**Reference**
9 M	No	6	0.9 fru/kg bw	^13^C-fru^(L1)^	42.9%	[70]^(2)^
9 F	No	6	0.9 fru/kg bw	^13^C-fru	43%	
8 M + 6 F	No	6	0.75 glu/kg bw	^13^C-glu^(L1)^	24.5%	[48]
8 M + 6 F	No	6	0.75 fru/kg bw	^13^C-fru^(L1)^	30.5%	
M + 3 F	No	6	0.5 fru/kg bw	^13^C-fru^(L2)^	56%	[49]^(2)^
3 M + 3 F	No	6	1.0 fru/kg bw	^13^C-fru	59%	
4 M + 4 F	No	3	0.9 fru/kg bw	^13^C-fru^(L3)^	38.5%	[52]^(2)^
7 obese F	No	3	0.9 fru/kg bw	^13^C-fru	34.9%	
8 type-2 (4 M)	No	3	0.9 fru/kg bw	^13^C-fru	31.3%	
10 M	yes	2	0.6 malt/min	^13^C-glu^(L4)^	81.7% (0.49 g/min)	[82]
10 M	yes	2	0.3 fru + 0.6 malt/min	^13^C-fru^(L4)^	62.0% (0.18 g/min)	
10 M	yes	2	0.5 fru + 0.6 malt/min	^13^C-fru	54.0% (0.27 g/min)	
10 M	yes	2	0.7 fru + 0.6 malt/min	^13^C-fru	52.0% (0.36 g/min)	
6 M	Yes	2	100 galactose	^13^C-galactose^(L1)^	23.7%	[60]
6 M	Yes	2	100 fru	^13^C-fru^(L1)^	38.8%	
6 M	Yes	2	100 glu	^13^C-glu^(L1)^	40.5%	
6 M	yes	2	100 fru	^13^C-fru(L1)	43.8%	[47]
6 M	yes	2	100 glu	^13^C-glu^(L1)^	48.1%	
6 M	yes	2	100 fru + 120 sucr	^13^C-fru	42.0% (42 g)	
6 M	yes	2	100 glu + 120 sucr	^13^C-glu	50.2% (50.2 g)	
18 M	yes	2	1.33 fru/kg bw	^13^C-fru^(L1)^	36.7% (35.7 g)	[62]
18 M	yes	2	1.33 glu/kg bw	^13^C-glu^(L1)^	57.2% (56.1 g)	
6 M	yes	2	100 fru	^13^C-fru^(L1)^	45.8%	[61]^(2,3)^
6 M	yes	2	100 glu	^13^C-glu^(L1)^	58.3%	
6 M	yes	2	50 fru + 50 glu	^13^C-fru + ^13^C-glu	73.6%	
6 M	yes	3	150 fru	^13^C-fru^(L2)^	38.0%	[50]
6 M	yes	3	150 glu	^13^C-glu^(L2)^	54.0%	
5 M	yes	2	1.33 fru/kg bw	^13^C-fru^(L4)^	51.0% (49 g)	[65]
5 M	yes	2	1.33 glu/kg bw	13C-glu^(L4)^	60.4% (58 g)	
5 M^3^	yes	2	1.33 fru/kg bw	^13^C-fru	37.5% (36 g)	
5 M^3^	yes	2	1.33 glu/kg bw	13C-glu	58.3% (56 g)	
6 M	yes	2	1.33 fru/kg bw	^13^C-fru^(L4)^	54.0% (53 g)	[64]^(3)^
6 M	yes	2	1.33 glu/kg bw	13C-glu^(L4)^	72.0% (70 g)	
6 M	yes	2	100 fru	^13^C-fru(L4)	54%	[55]
6 M	yes	2	100 glu	13C-glu^(L4)^	67%	
7 M	yes	3	140 fru	^13^C-fru^(L4)^	56%	[63]^(3)^
7 M	yes	3	140 glu	13C-glu^(L4)^	75%	
10 M	yes	2	1.0 fru/kg bw	^13^C-fru^(L2)^	43.0% (30 g)	[56]
10 M	yes	2	1.0 glu/kg bw	^13^C-glu^(L2)^	37.1% (26 g)	
8 M	yes	2	0.5 fru + 1.0 glu/min	^13^C-fru + ^13^C-glu^(L2)^	72.7% (1.09 g/min)	[68]^(3,4)^
8 M	yes	2	1.5 glu/min	^13^C-glu	50.7% (0.76 g/min)	
8 M	yes	2	1.2 sucr/min	^13^C-sucr^(L1)^	78.3% (0.94 g/min)	[67]^(4)^
8 M	yes	2	1.2 glu/min	^14^C-glu^(L1)^	58.3% (0.70 g/min)	
8 M	yes	2	0.6 sucr + 0.6 glu/min	^13^C-sucr + ^14^C-glu	70.8% (0.85 g/min)	
9 M	yes	2.5	1.8 glu/min	^13^C-glu^(L2)^	53.3% (0.96 g/min)	[69]^(4)^
9 M	yes	2.5	0.6 sucr + 1.2 glu/min	^13^C-sucr + ^13^C-glu^(L2)^	62.2% (1.12 g/min)	
9 M	yes	2.5	0.6 malt + 1.2 glu/min	^13^C-malt + ^13^C-glu^(L2)^	52.2% (0.94 g/min)	
8 M	yes	2	1.8 glu /min	^14^C-glu^(L1)^	38.7% (0.75 g/min)	[66]^(4)^
8 M	yes	2	0.6 fru + 1.2 glu/min	^13^C-fru + ^14^C-glu^(L1)^	64.4% (1.16 g/min)	

(1): hours = study monitoring hours, fru = fructose, glu = glucose, sucr = sucrose, malt = maltose; bw = body weight; M = male, F = female; type-2 = type-2 diabetes. In the reference column, (2) = with glucose infusion. (3) = non-fasting subjects. (4) = oxidation data of the 2nd hour or the last 1.5 hours. In the column of “Tracer”, superscripted L1 = labeled uniformly, L2 = naturally enriched, L3 = labeled at position 1, and L4 = ^13^C position not indicated.

The other studies in the Table 1 were conducted under conditions of exercise where workloads corresponded to 50-75% of max VO_2_ uptake. The oxidized amounts of ingested fructose ranged from 37.5% to 62.0%. Except in one study [60] which showed that fructose and glucose had similar oxidation rates (38.8% and 40.5%, respectively), the other studies all observed that glucose was oxidized faster than fructose under the exercise conditions [47,50,55,56,61-65]. A very interesting phenomenon noted is that when fructose and glucose are ingested together (including fructose-containing sucrose), the oxidation rates of the mixed sugars were faster than that of either one of them ingested alone at the same dosage. Adopo et al. reported that, given 100 g fructose, glucose, or fructose + glucose, 73.6% of the mixed sugars were oxidized while the data of fructose and glucose were 43.8% and 48.1% as ingested separately [61]. The series of studies by Jentjens and colleagues [66-69] also reported that fructose plus glucose or sucrose plus glucose consumed together were oxidized faster than glucose alone.

**Figure 2:**
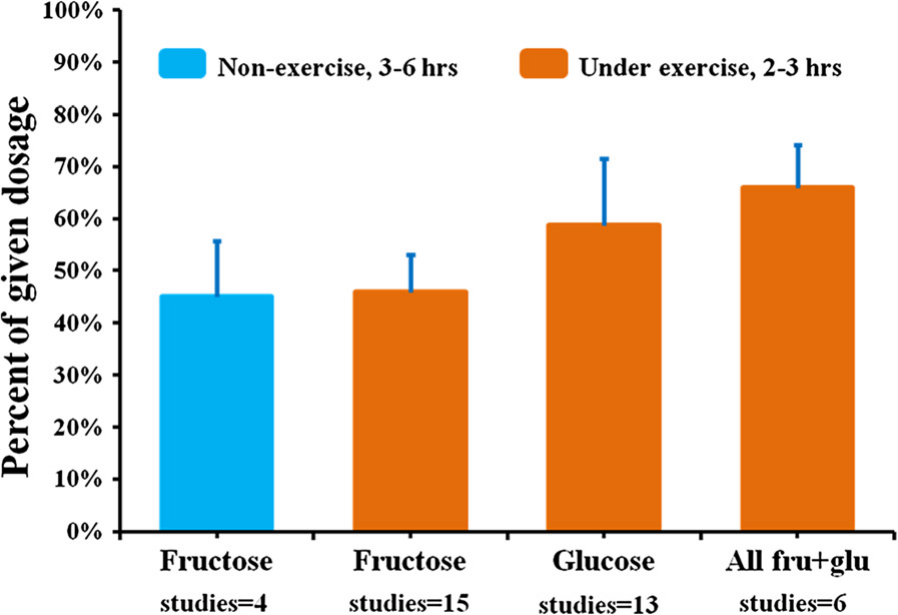
**Dietary fructose and glucose oxidation (in normal subjects, mean + SD).** On horizontal axis, ‘studies = number’ means that how many studies the bar data averaged from. In the Figure, 3–6 hours and 2–3 hours represent study monitoring period. The noted data variations between studies could be due to the differences of sugar dosages, tracer labeling forms, sugar administration methods, subject characteristics, and/or measurement errors. Also, the produced CO_2_ from labeled sugar oxidation can arise directly from sugar molecules themselves, or other compounds converted from the sugars, such as glucose, lactate, or fatty acid from fructose.

A summary of the sugar oxidation data is shown in Figure 2. The data of obese or diabetic subjects are not included in this figure. In non-exercise subjects, the mean of the oxidized fructose amount was 45.0% ± 10.7 (mean ± SD, range 30.5-59%) of ingested dose within a period of 3–6 hours. Under exercise conditions, this mean was 45.8% ± 7.3 (mean ± SD, range 37.5-62%) within 2–3 hours. When fructose and glucose are ingested in combination, either as fructose plus glucose, as sucrose, or as sucrose plus one of the 2 mono-sugars, the mean oxidized amount of the mixed sugars increased to 66.0% ± 8.2 (mean ± SD, range 52.2-73.6%). The oxidation data of glucose alone is 58.7% ± 12.9 (mean ± SD, range 37.1-81.0%).

Key points: 1) A significant amount of ingested fructose is oxidized by the body to produce energy. 2) Under resting conditions, fructose may be preferentially or similarly utilized to produce energy as glucose, and under exercise, glucose appeared to be more preferentially used to produce energy by the body. 3) When fructose and glucose are ingested together, the mixed sugars will be oxidized significantly faster than either one of the sugars ingested alone. And, 4) Fructose metabolism could be very different between normal and obese/diabetic subjects. A potential consideration with these oxidation studies is that with shorter time frames of measurement or incomplete oxidation and with only partial labeling, position of the isotope label can influence the rate of appearance of the isotope in the exhaled carbon dioxide (CO_2_). Temporal isotope appearance in CO_2_ can be altered if some of the fructose carbons are not completely oxidized in the time frame of measurement due to diversion to non-oxidative pathways.

#### Fructose-glucose conversion

The disposal pathway for fructose is not solely by direct oxidation, as some absorbed fructose will be converted to glucose. A number of studies have determined the extent of the conversion, which can only be clearly done using tracers. Table 2 tabulates the data from various studies with different experimental conditions. Tran and colleagues [70] studied the conversion of fructose to glucose as compared between men and women. After a 3 times ingestion of a fructose-containing beverage (3x0.3 g/kg bw), 37.4% of the fructose was converted to glucose in men during 6 hours. This value is significantly higher than the conversion rate of 28.9% observed in women. Similarly, using an equal fructose dosage, Paquot et al. [52] noted the conversion percent from fructose to glucose was 36.4% in 8 normal subjects (4 M + 4 F), which is comparable with Tran’s data. However, the conversion proportion appeared to be lower in obese and diabetic subjects (29.5% and 30.2%, respectively). In a dosing study monitored over a period of 6 hours, using 0.5 and 1 gram/kg bw, the conversion from fructose to glucose was reported to be 54% and 50.7% of given dosages, respectively [49]. Surmely et al. [71] infused fructose at 3 mg/kg bw per minute for the first 3 hours, followed by doubling the infusion dosage for the next 3 hours. It was noted that the subsequent higher infusion dose level somewhat slowed the fructose conversion percentage, 22% and 28% for high and low dose levels respectively. Under exercise conditions, Lecoultre et al. [51] reported that 29% of ingested fructose (96 g) is converted to glucose when a steady state of carbohydrate flux was reached (1.7-2 hrs from the beginning of study). With repeated administration to achieve a high dose level and under exercise, Jandrian et al. [50] reported that 55-60% of circulating glucose comes from fructose conversion during the latter half of the monitoring period. This data is similar to that observed by Delarue et al. [49] who reported the amount of glucose synthesized from fructose was 57% of overall glucose appearance in the circulation after an ingestion of fructose at dosage of 1 g/kg bw, while the subjects were not under exercise. These data suggest that 41% ±10.5 (mean ± SD, range 29-54%) of fructose can be converted to glucose within 2–6 hours after ingestion in normal non-exercise subjects. This conversion may be lower in women compared to men, and obese and diabetic subjects may also have lower conversion capability.

**Table 2:** **Conversion from Fructose to Glucose, Tracer Studies in Adults**^**(1)**^

**Subjects**	**Exercise**	**Hours**	**Fru dosages**	**Tracer**	**Blood glu (mmol/L)**	**Fru to glu conversion**	**Reference**
9 M	No	6	3x0.3 g/kg bw	^13^C-fru^(L1)^	5.94%^(2)^	37.4%	
9 F	No	6	3x0.3 g/kg bw	^13^C-fru	4.87%^(2)^	28.9%	[70]
4 M + 4 F	No	3	3x0.3 g/kg bw	^13^C-fru^(L3)^	5.2	36.4%^(3)^	
7 obese F	No	3	3x0.3 g/kg bw	^13^C-fru	5.3	29.5%^(3)^	
8 type-2 (4 M)	No	3	3x0.3 g/kg bw	^13^C-fru	7.7	30.2%^(3)^	[52]
3 M + 3 F	No	6	0.5 g/kg bw	^13^C-fru^(L2)^	4.56	54.0%	
	No	6	1.0 g/kg bw	^13^C-fru	4.66	50.7%	[49]
3 M + 3 F	No	0-3	3 mg/kg/min^4^	^13^C-fru^(L1)^	NA	28.0%^(4)^	
	No	4-6	6 mg/kg/min^4^	^13^C-fru	NA	22.0%^(4)^	[71]
7 M	Yes	2	96 g fru + 144 g glu	^13^C-fru^(L1)^	6.2	29%^(5)^	[51]
6 M	Yes	3	6x25 g	^13^C-fru^(L2)^	NA	55-60%^(6)^	[50]

For superscript numbers: (1), except Jandrain’s study [50], the subjects in the other studies were under glucose infusion; Hours = study monitoring hours, fru = fructose, glu = glucose; bw = body weight; M = male, F = female; type-2 = type-2 diabetes; NA = not available. (2), increases from baseline. (3), data were calculated based on reported parameters. (4), fructose administrated by infusion. (5), under steady state of carbohydrate flux. (6), percent of circulating glucose in the 2^nd^ half of study hours. In the column of “Tracer”, superscripted L1 = labeled uniformly, L2 = naturally enriched, and L3 = labeled at position 1.

For the conversion from dietary fructose to glycogen, data are very limited. Nilsson et al. [72] reported that a significantly higher amount of glycogen was determined in the liver (274.6 mmol glycosyl unit per kg wet tissue) after fructose infusion than that (76.2 mmol glycosyl unit) after glucose infusion; and no difference of glycogen increase in muscle was noted (23.0 and 24.4 mmol glycosyl units per kg after fructose and glucose infusions, respectively). Another fructose infusion study (non-exercise) by Dirlewanger et al. [73] noted that fructose stimulates total glucose output, glucose cycling and intrahepatic UDP galactose turnover, which was used as a marker for increased glycogen synthesis. Blom and colleagues [74] reported that dietary fructose could be about half as efficient as glucose or sucrose to replenish muscle glycogen after exercise. In that study, healthy young subjects exhaustively exercised on bicycle ergometers, and ingested 0.7 g/kg bw of fructose, glucose, or sucrose divided in 3 doses. The rates of glycogen synthesis in muscle corresponding to each sugar treatment were observed as 0.32, 0.58, and 0.62 mmol/kg per hour, respectively. The data indicate that energy status plays a role in how the body handles fructose distribution and conversion to glycogen. A more recent study reported that a part of dietary fructose was converted to glycogen based on surge of blood ^13^C-glucose concentration following a glucagon administration after 4 hours of ^13^C-labeled fructose intake (0.72 g/kg-bw) [75].

Although it was reported that a significant amount of fructose in the circulation could be used to produce glycogen in liver via first conversion to glucose, no isotope tracer studies were found to directly quantitate ^13^C-carbons from dietary fructose incorporated into glycogen in humans. Considering that most of absorbed fructose is extracted and metabolized in liver, the data from the fructose infusion studies noted above may not be representative for orally administered fructose.

Lastly, a number of studies using labeled glucose have examined how dietary fructose loads affect glucose production and disposal [76-78]. In these three studies, 6,6-deuterium labeled glucose was infused as a glucose metabolism tracer into male subjects after a 4–7 day fructose feeding, with fructose representing >25% energy in the diet. Results indicated that hepatic glucose production in normal subjects did not change [76], or had no effect on whole-body insulin-mediated glucose disposal [78]. Using a 2-step hyperinsulinemic euglycemic clamp, healthy offspring of Type 2 diabetics fed a high fructose diet exhibited higher fasting hepatic glucose levels compared to controls [77].

Key points: 1) Fructose is converted to glucose to variable extents, depending on exercise condition, gender, and health status. This interconversion occurs at the triose phosphate intersection of the glucose-fructose pathways. 2) A portion of fructose is incorporated into glycogen after conversion to glucose, but the extent is not known. 3) Fructose feeding has an effect on hepatic glucose production and whole body glucose disposal. And, 4) Fructose may be processed differently in obese population or population with higher diabetes risk.

#### Fructose-lactate conversion

Another significant and perhaps underappreciated metabolic pathway of dietary fructose is its conversion to lactate. Earlier tracer studies observed that blood lactate concentration was increased after fructose or fructose + glucose ingestion compared to that after glucose ingestion alone [56,66,68,79,80]. It was also observed that sucrose ingestion also caused a higher blood lactate response than did glucose [67,81]. However, no detailed data were reported to clarify how much of the ingested fructose was converted into the lactate in these studies.

Recently, Lecoultre et al. [51] conducted a tracer study in 7 men while under exercise. Within 100 minutes, 96 g fructose with 144 g glucose were co-ingested. The lactate conversion from ^13^C-labeled fructose was calculated using the parameters between 100 and 120 minutes when steady state of carbohydrate flux was assumed. As a result, 28% of fructose ingested was converted to lactate (35 micromol/kg-bw/min). Most of the converted lactate (25/28 or 89.3%) from fructose was oxidized mainly by working skeletal muscle (31 micromol/kg-bw/min). The non-oxidative fructose disposal was 0.52 grams per minute accounting for about 40% of the fructose ingested. The rate of appearance of glucose from fructose conversion was 19.8 micromol/kg-bw/min or 29% of the fructose dose. The authors also indicated that the increased lactate production and oxidation would be an essential explanation of faster oxidation of fructose + glucose co-ingested than glucose ingested alone.

In the tracer study by Rowland and colleagues [82], blood lactate concentration changes were compared in 10 men under exercise using oral test solutions of ^13^C-labeled glucose + ^14^C-labeled fructose (at 0.6 g/min glucose + 0, 0.3, 0.5, or 0.7 g/min fructose). During the 2-hour study period and compared to glucose alone, plasma lactate amount increased 31% and 24% under the glucose + fructose ingestions at 0.6 + 0.5 g/min and 0.6 + 0.7 g/min, respectively. However, the study did not indicate conversion percentage from the labeled fructose or glucose dosages.

Key points: 1) Clearly, a significant amount of fructose can be converted to lactate, but quantitative metabolic data of dietary fructose to lactate conversion is very limited. The effects of fructose dose, administration method, physical activity, and subject characteristics on fructose-lactate metabolism remain to be further studied. 2) Labeling patterns of isotope tracer in fructose will have an influence on the measured isotope appearance in lactate, if the studied sugar is not uniformly labeled.

#### Fructose-lipids conversion

A significant number of clinical studies have been performed to investigate the influence of fructose intake on blood triglyceride (TG) concentrations. However, tracer studies aimed at revealing metabolic conversion from labeled fructose carbons to TG are extremely limited. In contrast to the conversion from fructose to glucose, the metabolic pathway from fructose to TG conversion can be much more complicated due to the complex distribution and diversity of blood lipid compositions in the body. De novo lipogenesis from sugars can occur in the liver and end up as packaged VLDL TG and/or as intrahepatocellular lipids. There are currently no convenient methods to quantitate overall DNL and intrahepatic lipid deposition. The fractional contribution of sugars to de novo lipogenesis and VLDL TG are commonly determined using tracer enrichment data of blood samples. The time periods of liver de novo lipogenesis from sugars and the factors influencing it are not completely understood, and are impacted by the concentrations and tracer characteristics of the various substrates drawn from lipid precursor pools. De novo lipogenesis may also occur in adipose tissue or muscles, but there are no adequate methods available to quantitate it. A more expansive discussion of de novo lipogenesis and methodological considerations is an appropriate subject for a separate review.

Perhaps because of these difficulties, only two tracer studies were found that investigated conversion of labeled dietary fructose carbons into plasma lipids. Chong et al. [48] studied the effect of fructose on postprandial lipidemia in fourteen adults (8 men) who were orally administrated ^13^C-labeled fructose or ^13^C-labeled glucose at a dose of 0.75 g/kg bw, together with an ^2^ H-labeled oil mix (85% palm oil and 15% sunflower oil) at 0.5 g/kg bw. Blood lipid changes were monitored in a 6-hour period. It was observed that plasma TG concentration rose more significantly after fructose ingestion (from baseline 1240 μmol/L (≈110 mg/dl) to its plateau of 2350 μmol/L (≈208 mg/dl)) than that after glucose ingestion (from baseline 1240 μmol/L to its plateau of 1700 μmol/L(≈150 mg/dl)). However, the concentration increases of ^13^C-enriched TG-fatty acids and TG-glycerol from the labeled fructose in the S_f_ 20–400 lipid fraction (including VLDL) were very small within the monitoring period. The plateau value of ^13^C-palmitate concentration was about 0.022 μmol/L (≈0.002 mg/dl), ^13^C-myristate was about 0.0015 μmol/L (≈0.0001 mg/dl), and ^13^C-TG-glycerol was about 1.4 μmol/L (≈0.124 mg/dl), suggesting that fructose carbons were not substantially transferred into plasma TG molecules during the time period monitored. The authors indicated that the lipogenic potential of fructose seems to be small, since the results showed that only 0.05% and 0.15% of fructose were converted to de novo fatty acids and TG-glycerol at 4 hour, respectively. The reported data should be viewed in the context of the 4-hour time period and whether further conversion would be observed at extended times was not illustrated. It was observed by Vedala and colleagues [83], using labeled fatty acids, acetate and glycerol as precursors, that a meaningful portion of de novo synthesized triglyceride would appear in blood at later times, and rates of this delayed secretion were significantly different among normal, hypertriglyceridemic, and diabetic subjects. However, this study did not specifically measure fructose conversion using labeled sugars.

In another study, Tran et al. [70] reported that ^13^C-labeled fructose consumption at 3x0.3 g/kg body weight caused a small but significant increase of ^13^C-enrichment in VLDL palmitate in 8 men compared with that found in 9 women (no increase) during a 6-hour monitoring period. However, compared to baselines, plasma TG and non-esterified fatty acid concentrations decreased 5.3% and 32.9% in men and 3.3% and 24.4% in women, respectively. The data indicate that the conversion from fructose to fatty acid occurred, however, no blood lipid concentrations increased. Although the authors reported that 42.9% and 43% of the ingested fructose was oxidized and 37.4% and 28.9% was converted into glucose in men and women during the 6-hour monitoring period, conversion rate or percent from fructose into fatty acid or triglyceride was not reported. This study also noted that men processed dietary fructose differently than women, and the given fructose lowered postprandial plasma lipids. It was discussed that although fructose is a potent lipogenic substrate, the observed fat synthesis arising from fructose carbons appeared to be quantitatively minor compared with other pathways of fructose disposal, but it may nevertheless have a significant impact on plasma and tissue lipids. In this same study, respiratory quotient (RQ) measurements found differences between genders, with male subjects increasing their RQ by 3% and females maintaining theirs. This data suggest that the increase of blood TG frequently observed in men compared to women after high dose fructose ingestion could be due to fat sparing during energy utilization.

There are several studies which used labeled acetate, administered by intravenous infusion as a precursor of lipid synthesis, to assess the fructose stimulation of de novo lipogenesis (DNL). This technique uses the approach of Mass Isotopomer Distribution Analysis (MIDA) to estimate the infused subunit (acetate) appearance in newly synthesized fatty acids and further predict the effect of dietary fructose on fractional DNL. The advantages and limitations of the method were well reviewed by Hellerstein in 1996 [84]. Parks et al. [85] investigated the influence of fructose-containing drinks on blood lipid changes using infused ^13^C-acetate. Six healthy subjects were randomly administrated 86 grams (mean) of glucose, glucose + fructose (50:50) or glucose + fructose (25:75) in drinks by a crossover-designed trial. Four hours after fructose ingestion, a standard lunch was consumed. Compared to glucose, more palmitate synthesis in triglyceride-rich lipoprotein (TRL) TG was noted after fructose-containing drinks, but not after the lunch. No significant differences were observed for TRL-TG concentrations between glucose and fructose-containing drink arms after baseline correction. Plasma TG concentration was decreased after glucose preload and stayed constant after fructose-containing drink preloads. Following the lunch, TG concentrations increased for all treatments. The authors reported that the after lunch TG-AUC data from fructose-containing drink treatments were significantly larger than that of glucose drink treatment. However, this AUC data was calculated over the entire study time period. Due to the negative TG rise during the glucose preload phase, the difference between the glucose and fructose arms was accentuated.

Similarly, in Stanhope and colleagues’ study [86], ^13^C-labeled acetate infusion was used to measure fractional DNL in a 10-week intervention involving 18 overweight or obese subjects consuming either glucose (n = 8) or fructose beverages (n = 10) delivering 25% of daily energy. The percent changes of fractional hepatic DNL were not significantly different from baseline following 9-week glucose consumption for both fasting and postprandial measurements. In the fructose beverage group, the percent changes of fractional hepatic DNL were also not significantly different between baseline and following 9-weeks for fasting data, but were significantly increased for postprandial data (2-7% during 11-hour monitoring). The actual amount of the DNL was not reported.

Faeh et al. conducted a shorter term crossover study using a 6-day intervention [78]. Seven men were fed hypercaloric (+800-1000 kcal/d) diets, with the additional 25% of energy provided through a fructose solution. Fractional hepatic DNL was measured via ^13^C-labeled acetate infusion. The % changes from baseline for plasma TG and hepatic DNL were found to be significantly increased for the hypercaloric fructose diet compared to isocaloric control diets. The authors noted that the results could not truly differentiate the effects of the high-fructose intake per se and that of the total carbohydrate energy overfeeding. This study also found that fish oil added to the diets containing fructose attenuated this hyperlipidemic response somewhat [78].

Clearly, the 3 studies discussed above [78,85,86] assess effects of dietary fructose with or without over energy intakes on the utilization of acetate in the circulation, which is designed to feed directly into lipid synthesis. In humans, acetate concentrations in blood are fairly low. As indicated in the Human Metablome Database [87], normal blood concentrations of acetate are 41.9 ± 15.1 (SD) μmol/L in adults aged 18 years and over. For earlier data, Richards et al. [88] reported in 1976 that the normal value of blood acetate was 25 ± 2 μmol/L. Beyond alcohol consumption, common dietary intakes have no or limited influence on blood acetate concentration [89,90]. In the studies of Parks, Stanhope, and Faeh, acetate was constantly infused at 0.5-0.55 g/hr (about 7000 μmol/hr) for 25, 26 and 9.5 hours, respectively. Although the data of blood acetate concentration were not reported in those studies, it would be important to determine whether the acetate infusion significantly raised blood acetate concentrations such that this could have a meaningful impact on metabolic response to the fructose challenge. The coexistence of the infused acetate and intermediate metabolites of fructose, including regulatory elements of citrate, malate, and lactate, could prime the pathway of DNL. As detailed above, dietary fructose (up to 25% of daily energy and 3 g/day-kg in these studies) can metabolically be converted into lactate and further result in blood lactate concentration increases. Beynen and colleagues’ hepatic cell study [91] indicated that lactate and acetate both stimulate fatty acid synthesis, and lactate can induce activation of acetyl-CoA carboxylase, a key enzyme for fatty acid synthesis. Thus, the meaning of stimulation of de novo lipogenesis observed from use of infused intermediate metabolite tracer and its interaction with the sugars studied should be considered carefully, along with the study methodologies being validated for the dosage of infused tracer. Additionally, how the infused acetate can truly represent intrahepatocellular acetyl-CoA pool is another key point to be clarified.

Key points: 1) The above tracer studies indicate the complex relationship between dietary fructose and lipid synthesis. The observed increases in plasma TG and DNL in these studies can arise from both increased lipid synthesis and decreased lipid clearance, and the relative contributions were not addressed in any detail. And, 2) The intake levels, health status, and gender of subjects are all important factors influencing sugar-lipid relationships. The influence of fructose consumption on plasma lipids and de novo lipogenesis remains controversial and understudied.

#### Influence of exogenous sugars on utilization of endogenous energy sources

After sugar ingestion, body utilization of energy sources will change. As exogenous carbohydrate is used as a fuel source, the oxidation rates of stored energy, typically, endogenous carbohydrates and fat, will decrease. The extent of the decrease is usually driven by ingested sugar type, intake amount, and status of body energy need (such as vigorous exercise or screen watching). Under exercise, glucose is more likely to be preferentially oxidized than fructose, and this scenario will go in the opposite direction under a resting state. Although data are limited related to detailed shifting of energy sources under different conditions, some studies using subjects under exercise may provide a basic concept of energy source shifting after sugar ingestion. Jentjens and colleagues conducted a series of studies [66-69,80] using exercise subjects under somewhat comparable conditions, and reported some data related to the energy source shifting. The subjects were given drinks containing glucose, sucrose, glucose + fructose, or glucose + sucrose at dosage 0 (control), 1.2, 1.5, 1.8, or 2.4 g/min and under exercise workloads around 50% VO2 max uptake. For controls (0 gram of sugar intake), the oxidation rates of fat and endogenous carbohydrate were between 0.77-0.95 g/min and 1.43-1.85 g/min, respectively. Compared to the control, glucose-containing drinks decreased fat oxidation rates by 21.6-41.7% (calculated based on reported data) and endogenous carbohydrate oxidation rates by 8.5-31.5%, except that one of the 5 studies noted endogenous carbohydrate oxidation rates increased (3.8% for medium and 14.1% for high glucose intake). For fructose-containing drink arms, either for glucose + fructose, sucrose, or glucose + sucrose, the fat oxidation rates were lowered by 19.5-47.4% and endogenous carbohydrate oxidation rates were lowered by 13.0-31.6%. These percent decreases appeared to be positively correlated to the sugar intake levels and the ratios of fructose in the mixed sugar drinks. The other two studies [60,79] with similar settings as Jentjens and colleagues’ work also reported comparable data of decreasing fat and endogenous carbohydrate oxidation after sugar preloads.

Key points: 1) Together with other sugar inter-conversion data and the RQ data of Tran et al. [70], the shifting of energy sources after sugar ingestion may indicate that the utilization of exogenous and endogenous energy is closely regulated according to the energy balance of body. 2) Beyond specific health and physiological conditions, physical activity, over energy consumption, dietary macronutrient composition, and other lifestyle factors would also play critical roles in the body’s utilization of dietary sugars. In view of these factors, how energy is quantitatively balanced with fructose loading is an area yet to be delineated.

## Summary

**Figure 3:**
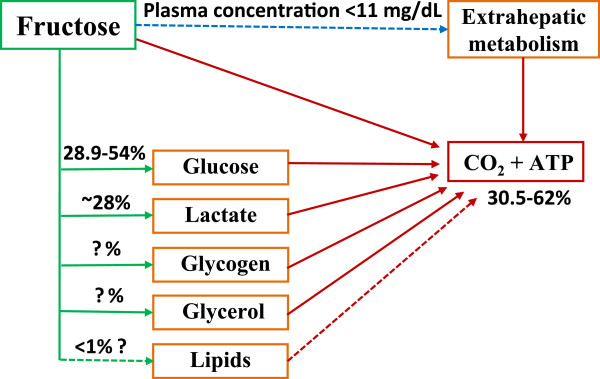
**Metabolic fate of dietary fructose carbons.** The data are obtained within study periods less than or equal to 6 hours. After 50–150 gm fructose ingestion, the peak of fructose concentration in plasma would be between 3–11 mg/dL. The percent data above arrow lines or under box are the estimated amounts of ingested fructose doses via the pathway, and the question mark represents that the data remain to be further confirmed. The dash-line represents presumably minor pathways.

Figure 3 summarizes the major metabolic fates of dietary fructose based on the data obtained from the reviewed isotope tracer studies. The mean oxidation rate of dietary fructose was 45.0% (ranged 30.5-59%) of ingested doses in normal subjects within a period of 3–6 hours. With exercise conditions, the mean oxidation rate of fructose came to 45.8% (ranged 37.5-62%) within 2–3 hours. When fructose was ingested together with glucose, the mean oxidation rate of the mixed sugars increased to 66.0% (ranged 52.2-73.6%) under similar exercise conditions. Secondly, the mean conversion rate from fructose to glucose was 41% (ranged 29-54%) of ingested dose in 3–6 hours after ingestion in normal non-exercise subjects. This value may be higher in subjects under exercise. The conversion amount from fructose to glycogen remains to be further clarified. Thirdly, at short time periods (≤ 6 hours), it appeared that only a small percent of fructose carbons enter the pathway of liponeogenesis after fructose ingestion. The hyperlipidemic effect of dietary fructose observed in both tracer and non-tracer studies may involve other metabolic mechanisms and this could relate to energy source shifting and lipid sparing. Lastly, fructose can be catabolized into lactate and cause an increase of blood lactate concentrations. Approximately a quarter of ingested fructose could be converted into lactate within a few of hours and this is a means to release fructose-derived carbons from the liver for extrahepatic utilization. Even though the reviewed tracer studies may not be fully representative of real-life diets and the obtained data are limited, this review provides a basic outline how fructose is utilized after it is consumed by humans.

## Competing interests

The authors are employed full time by Archer Daniels Midland Company (ADM). ADM is a major oilseed and grain commodity processor and produces, among other products, fructose-containing sweeteners.

## Authors’ contributions

The two authors, SZS and MWE, have made similar contributions to the review. Both authors have read and approved the final manuscript.
